# Identifying individual polar bears at safe distances: A test with captive animals

**DOI:** 10.1371/journal.pone.0228991

**Published:** 2020-02-13

**Authors:** Jouke Prop, Arnstein Staverløkk, Børge Moe

**Affiliations:** 1 Arctic Centre, University of Groningen, Groningen, The Netherlands; 2 Norwegian Institute for Nature Research (NINA), Trondheim, Norway; The Cyprus Institute, CYPRUS

## Abstract

The need to recognise individuals in population and behavioural studies has stimulated the development of various identification methods. A commonly used method is to employ natural markers to distinguish individuals. In particular, the automated processing of photographs of study animals has gained interest due to the speed of processing and the ability to handle a high volume of records. However, automated processing requires high-quality photographs, which means that they need to be taken from a specific angle or at close distances. Polar bears *Ursus maritimus*, for example, may be identified by automated analysis of whisker spot patterns. However, to obtain photographs of adequate quality, the animals need to be closer than is usually possible without risk to animal or observer. In this study we tested the accuracy of an alternative method to identify polar bears at further distances. This method is based on distinguishing a set of physiognomic characteristics, which can be recognised from photographs taken in the field at distances of up to 400 m. During five trials, sets of photographs of 15 polar bears from six zoos, with each individual bear portrayed on different dates, were presented for identification to ten test observers. Among observers the repeatability of the assessments was 0.68 (SE 0.011). Observers with previous training in photogrammetric techniques performed better than observers without training. Experience with observing polar bears in the wild did not improve skills to identify individuals on photographs. Among the observers with photogrammetric experience, the rate of erroneous assessment was on average 0.13 (SE 0.020). For the inexperienced group this was 0.72 (SE 0.018). Error rates obtained with automated whisker spot analysis were intermediate (0.26–0.58). We suggest that wildlife studies will benefit from applying several identification techniques to collect data under different conditions.

## Introduction

When investigating life history processes in wildlife, the ability to identify individual animals carries great weight. Therefore, methods that facilitate the recognition of individuals are numerous, and continuously improved and extended to integrate rapidly emerging technologies [1] and address new research questions [2]. One line of approach is to catch animals and apply artificial markers, such as individually coded leg rings, neck bands, or passive integrated transponder tags [3]. Another approach is to use natural markers, which are characteristics that are shared by all animals within a species but for which precise individual phenotypic expression is unique [4,5]. Examples of natural markers include spotted coat patterns in felids [6], notches in the fins of whales and dolphins [7], and the black-and-yellow bill markings in swans [8]. Usually, the animals are photographed in the field, which enables the researcher to process and analyse the photographs at a later stage [1]. Natural markers are bound to lack the potentially very high accuracy and ease of human-deployed artificial markers [9], but using natural markers has the significant advantage that study animals do not need to be approached, caught or handled [10]. Problems associated with catching include risk of injury to the animal or researcher [11–13], disease transfer [14], disturbance [15,16], and physiological stress [17–19]. Moreover, in many cases the segment of a population caught and marked does not necessarily contain all the target individuals that are relevant for a particular study.

The suitability of natural markers reaches its limitations, however, when the amount of detail that needs to be distinguished conflicts with the proximity to which the animal can be safely approached. The relatively coarse-grained details in coat patterns of giraffes or zebras may be easily distinguishable at regular working distances [20,21]. However, detecting whisker spot patterns may be more challenging, depending on the study species, working conditions, and equipment used. At increasing working distances (1–2 m in Australian sea lions *Neophoca cinerea* [22], 10–30 m in lions *Panthera leo* [23], up to 50 m in polar bears *Ursus maritimus* [24]), detecting whisker spot patterns may approach the edge of what is possible. Even if it is possible to approach wildlife at sufficiently short distances, it may not be desirable to do so. Habituation by wild animals, especially carnivores, to human proximity may lead to undesired effects with potentially lethal consequences for the animal or humans [25,26].

For the reasons outlined above, an appropriate natural marker should be distinguishable in photographs taken from sufficiently far distances to avoid disturbance or other harm to the study animal. There may be a trade-off between keeping a safe distance and the ease or efficiency of processing the images. It may therefore be advantageous to deploy various techniques even within a study of the same species. The choice of technique(s) will depend on the conditions during the encounter, which are primarily set by the achievable or desirable distance to the study animals.

As a top predator of the arctic sea ice, the polar bear is an iconic species that is suffering from the consequences of global warming [27,28]. To comprehend current problems in arctic ecosystems associated with rapidly decreasing sea ice, extensive research is urgently needed on the behaviour and ecology of polar bears, in addition to population monitoring [29,30]. With their evenly coloured and extremely thick fur, polar bears lack the obvious characteristics that would otherwise enable researchers to identify study animals. The pattern of whisker spots, which can be derived from photographs, has been found to be a reliable natural marker in polar bears [24]. However, to distinguish the precise pattern of these spots, a close-up view of the head within 50 m is required [24]. Near approach is possible where the bears are used to human presence and when observers are safely seated in large vehicles [31]. Over much of their range, however, polar bears are shy of humans, and the observation distances that do not cause disturbance will usually be in the range of hundreds of meters [32].

In this study, we explored alternative ways of identifying polar bears using a variety of cues that could be helpful in distinguishing individuals from a further distance than that needed for whisker spot analyses [33]. Anderson et al. [24] claimed to be able to identify individual polar bears based on scar patterns, gender, and body size and shape but did not provide evidence on the accuracy of this method. To identify individuals, we used a broad range of physiognomic features, which were selected based on extensive recognition trials of polar bears in the wild. The features include shape of the body and head, the occurrence of scars, patterns of hair strands on the head, and the pattern of dark patches on the muzzle. To experimentally test the reliability of this method, we collected sets of photographs of individual polar bears, with each individual being portrayed at three different dates, from various zoos in Europe and North America. A similar level of detail in photographs can be achieved when photographing polar bears at distances ranging in the hundreds of meters in the field by commonly available optics, including high-magnification telescopes. The identity of the polar bears was known to the experimenter but not to the test observers. The task of the test observers was to distinguish the bears in the photographs in a series of successive trials by visual matching. Test observers were recruited from among those with extensive experience with polar bears in the wild, those trained in photogrammetric techniques, and those without previous relevant experience. The bears were also identified following the method developed by Anderson et al. [34], which is based on automated analysis of individual-specific patterns of whisker spots. Specifically, we aimed to (1) test the reliability of identifying polar bears individually based on physiognomic characteristics, and assess the importance of analytical training or acquaintance with polar bears, and (2) compare the reliability of this method with results obtained by analysis based on whisker spot pattern.

## Methods

Photographs of 15 different polar bears were obtained from six zoos in Europe and North America (Table 1). Zoo managers and polar bear care takers were requested to take photographs of each polar bear on three different occasions. The goal was to have photographs collected at a time scale of weeks or months, corresponding to the length of a typical field season in the Arctic. In practice, the occasions were separated by an average of 25 days (range 4–205 days), with 75% of successive occasions occurring within 15 days. One of the individuals was photographed on only one occasion, which means that, in total, 43 collections of photographs taken at a single occasion (called “sets” hereafter) were available. Photographers were further asked to take pictures of active bears (not sleeping or lying on the ground) and to use a camera with a suitable zoom lens, such that the bear covered 50–90% of the image. This resulted in images in which the body of the polar bears covered 0.3–10 megapixels (on average 4.0 megapixels), which corresponded to the resolution that can be obtained when photographing polar bears in the wild at a distance of 200–400 m with a suitable camera system. A bear covering 0.5 megapixels is sufficient to distinguish the details used in this study, as long as the photographs are in focus and sharp. Most but not all photographs met the minimum quality requirements. During some occasions, the light conditions at the time of shooting were poor and resulted in blurry or grainy photographs, impairing the visibility of details. Nevertheless, poor photographs were included in the identification process. To further simulate field conditions, photographers in the zoos were encouraged to take pictures at different times of the day and, if possible, to select days with various weather conditions. Photographers were further asked to portray the bears from a front view of the top of the head, front view right, front view left, left view and right view (S1 Fig). Before initiating the trials, any cues that could help reveal the identity of the polar bears were removed from the photographs. This was done by erasing the background and by removing all embedded information on time of picture, camera type, and GPS coordinates.

**Table 1:** Overview of the number of male and female polar bears from six zoos featured in the trials.

	Male	Female	Total occasions
Aalborg Zoo, Denmark	0	1	3
Copenhagen Zoo, Denmark	1	1	5^a^
Lincoln Park Zoo, Chicago, IL, USA	1	1	4^b^
Ouwehands Dierenpark, Rhenen, The Netherlands	0	2	6
Point Defiance Zoo & Aquarium, Tacoma, WA, USA	2	0	6
Skandinavisk Dyrepark, Kolind, Denmark	4	2	18
Total	8	7	42

Each individual bear was recorded during three occasions (see notes for exceptions).

^a^) One occasion missing for female.

^b^) Two occasions missing for female.

### Experimental setup

For each of the five trials, the test observers were presented with 15 sets of photos. Sets were composed of an average of 13 photographs (range 7–20), each featuring the same individual. The task of the test observers was to judge correspondence in identity among sets. The sets were chosen randomly without replacement, resulting in 10 or 11 (average 10.4) different individuals per trial. Trials were treated independently of each other, such that the same set of photographs could appear in multiple trials. Nevertheless, all comparisons concerned unique pairs of sets. The test observers knew that all photographs within a set were of the same individual and that the number of sets of the same individual was three at the highest, but the total number of different bears in the tests remained undisclosed. Information on the (relative) date of collection was also not provided. To facilitate comparisons of photographs within and among sets, photographs were ordered depending on the angle of the bears’ heads: bear facing far left (or left backward), facing towards the camera, and facing far right. If needed, the quality of photographs was improved by adjusting contrast. To test the effect of experience on the ability to identify individuals, four types of test observers were recruited depending on any combination of (a) previous extensive experience in observing polar bears in the wild and (b) trained in identifying objects (not necessarily polar bears) in photographs. Sample sizes of the types (experience with polar bears and with or without photogrammetric techniques, no experience with polar bears and with or without photogrammetric techniques) were 2, 2, 2 and 4 test observers, respectively. The net time to process a single trial was 1–2 days, and processing all trials was spread over a period of 2–5 weeks.

### Processing photos

**Fig 1:**
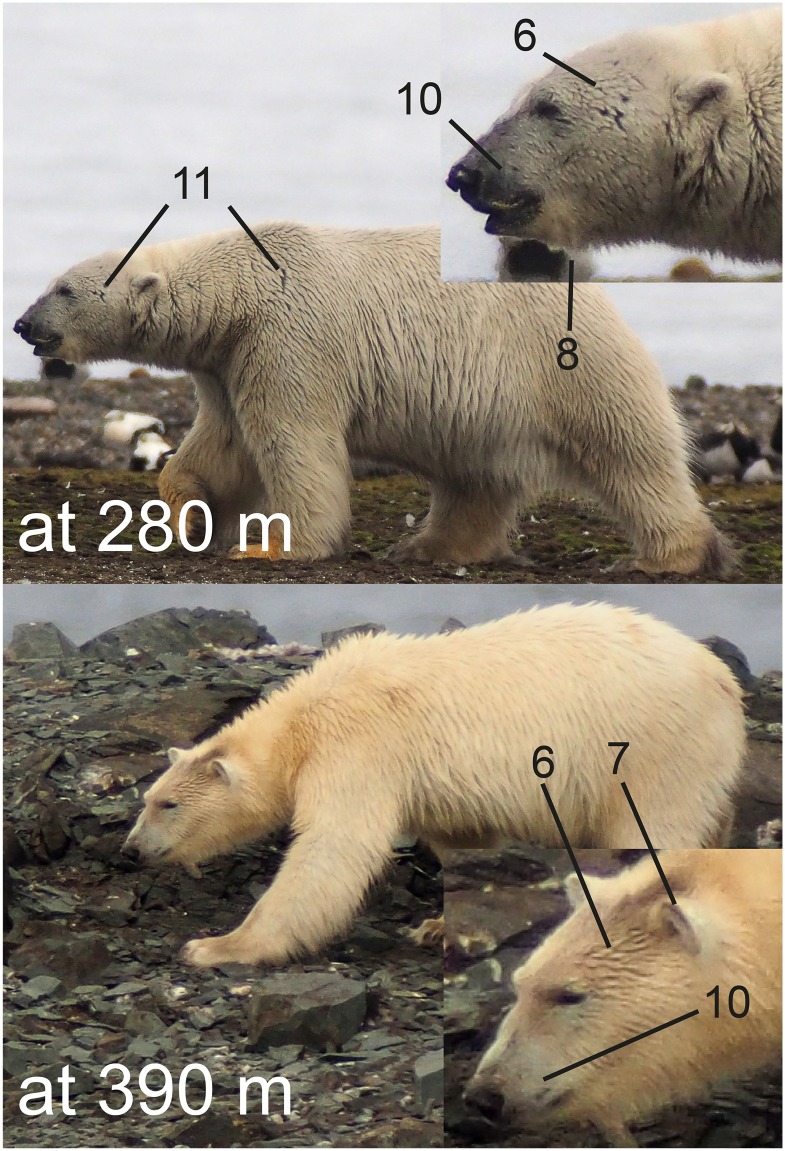
Some features used to distinguish individual polar bears. The numbers refer to the list in Table 2. The distance between camera and bear is indicated. The upper photo shows a male, the lower a female. Photographs are from a field study site on Nordenskiöldkysten, Svalbard, using a camera (Olympus OM-D E-M5) attached to a telescope (Swarovski STX 95) at 30× magnification.

**Fig 2:**
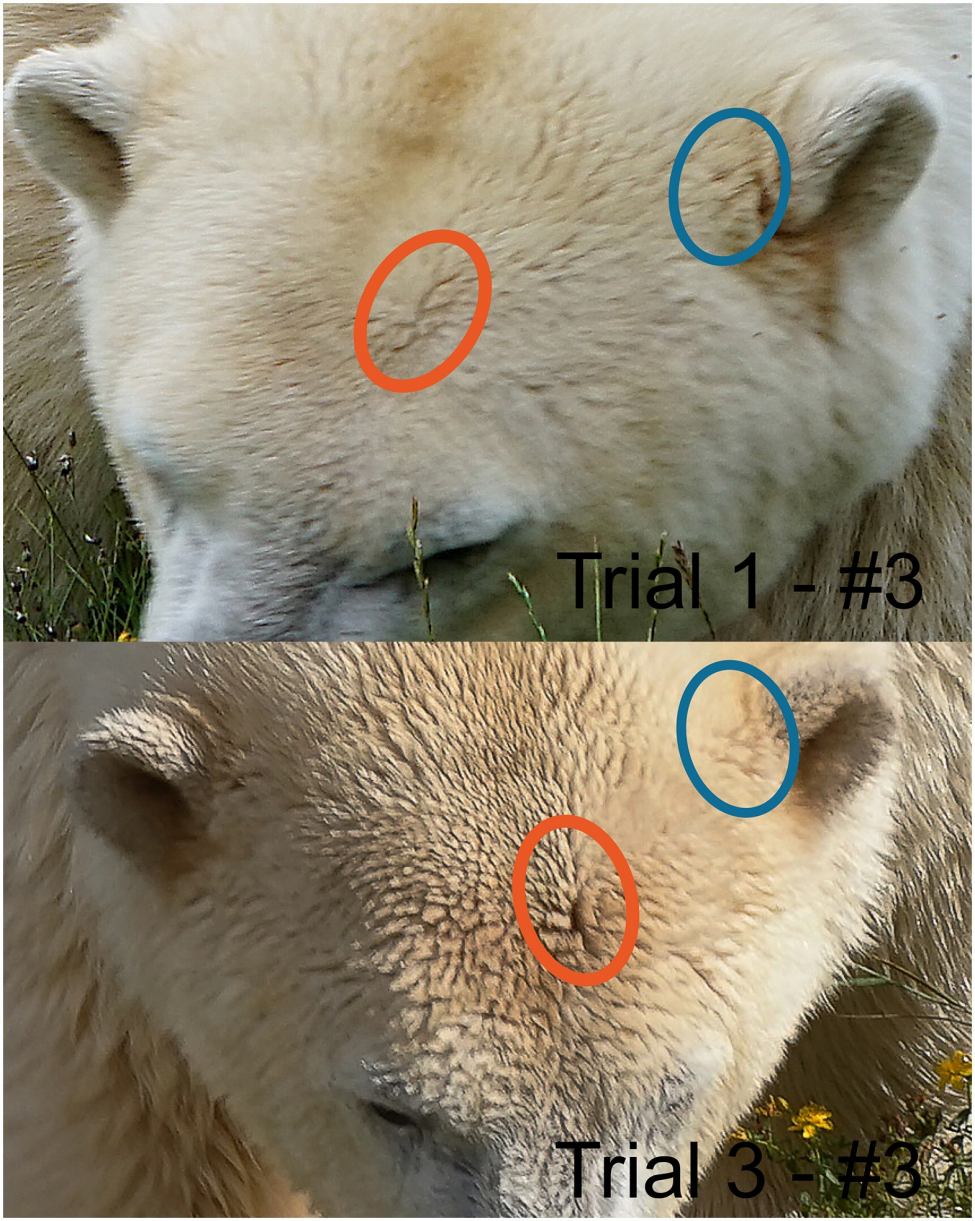
Example of hair patterns used to identify individual polar bears. Similarities in patterns helped to identify individuals. These photographs were taken 15 days apart and were of the same individual (“Ilka”, Skandinavisk Dyrepark, Denmark).

For each set, bears were classified according to a list of features (Table 2), which included information on posture, head shape, body condition, pattern of hair over the body and on the head. What these generic features have in common is that they are distinguishable in wild polar bears at distances of up to 400 m (Fig 1). Furthermore, test observers were asked to identify any ad hoc features that an observer might find useful as a natural marker. Details of the head were mapped on a standard sheet [4] with head profiles (left side, right side, and top of the head), including the pattern of dark spots between nose and lips, and the pattern of grey tones on the muzzle, scars or wounds, and hair patterns (see Fig 2 for an example). By pairwise comparisons, sets were screened for corresponding features. In the case that a match was suspected, photographs of the two sets were compared side by side and closely inspected to confirm (or reject) that the same individual was involved. These assessments resulted in the preliminary ratings, composed of a matrix of all sets against each other in which observers coded their ratings as “D” or “S” (when sets were thought to be from different or the same individuals, respectively). Trials were processed consecutively, and after completing the five trials the test observers were given the opportunity to reconsider assessments. This was done to allow for the effects of any experience built up during the trials, resulting in the final ratings used in the analyses.

**Table 2:** Physiognomic features used to distinguish polar bears.

Features	Categories
1) Posture/build	Massive, heavy, slender
2) Head shape	Broad, slender, pointed
3) Body condition	Scale of 5 (skinny to fat, Stirling et al. [35])
4) Belly size	Scale of 3 (flat to bulging)
5) Teeth (in particular canines)	Damaged or not
6) Hair pattern on top of head and muzzle	Shape of strands
7) Hair coverage of outer ear	Fully covered or not
8) “Beard”	Present or not
9) Throat fold	Present or not
10) Pattern of dark patches on muzzle	
11) Pattern of scars or wounds on head	
12) Pattern of scars or wounds elsewhere	
13) Any other identifying features	

### Statistical analyses

The experiment was structured as a hierarchical design, in which 15 sets of photographs were presented in five consecutive trials. All possible pairs among the sets amounted to 5 × 15 × (15 − 1) / 2 = 525 comparisons, each with a unique comparison ID. All comparisons were rated by the ten observers. Thus, comparisons were nested within trials, and observers were crossed by comparisons (data in S1 Dataset). Each pairwise comparison resulted in one of four possible outcomes [36], depending on the observer’s rating and the similarity class (whether bears were the same or different individuals). In those cases that bears were the same, they were either correctly identified (true positive, TP) or they were misidentified as being different (false negative, FN). The false negative rate is the proportion of misidentifications, or *FNR* = (∑*FN*) / (∑*TP* + ∑*FN*). Likewise, when bears were different, they were either correctly identified (true negative, TN) or they were misidentified as being the same (false positive, FP). The false positive rate is *FPR* = (∑*FP*) / (∑*TN* + ∑*FP*). The error rate ER follows from the Euclidian distance between FPR and FNR as *ER* = √(*FPR*^2^ + *FNR*^2^). For calculation purposes, the observers’ ratings were recoded into a variable “outcome”, coded as 0 when the ratings were correct and 1 when the ratings were incorrect. In this way, “outcome” averaged by similarity class estimates FPR and FNR.

The statistical analyses were performed with the software R [37]. The observations were analysed by generalized linear mixed-effects models, glmer in the R-package lme4 [38] adopting a binomial distribution and logit link function. To account for the structure of the experiment, observer and comparison ID nested within trial were treated as random factors. To explore the repeatability among observers [39], the ratings were modelled in a random effects model. Repeatability, defined as the variance due to random effects as proportion of total variance [39], was obtained by the function rptBinary in the R-package rptR [40], with the built-in functionality to estimate the standard error by parametric bootstrapping (1000 times in our case).

Outcome was subjected to mixed-effects modelling with two fixed factors: similarity class and a variable representing the four levels of observers’ experience. It was not possible to analyse effects of the four levels of experience, similarity class and their interaction simultaneously as parameters could not be estimated due to convergence problems. Therefore, we followed a two-step approach by first exploring main effects of experience and similarity class, and subsequently testing any interaction effects. Testing for differences among the experience types was by post-hoc pairwise comparisons in the R-package emmeans [41] with Tukey adjustment. Significance of fixed factors was under the assumption that the coefficient’s estimates are normally distributed (z-test). As models generated by glmer do not provide a way to calculate standard errors of predictions, standard errors and 95% confidence intervals were estimated by bootstrapping using the R-package boot [42], based on 1000 replicates.

To evaluate the performance of the observers against a random process, the ratings were compared with the outcome of 1000 simulations for each of the five trials (S1 File for an example script). Starting with the sets in the original order of the experiment, a matrix comparing all sets to each other (rated as “different” or “same”) was generated. Subsequently the order of the sets was randomized resulting in a new matrix with ratings in random order. The two matrices were compared cell-by-cell which resulted in corresponding measures of agreement (TP, TN, FP or FN). From the simulated FPR and FNR the means were calculated, and the 95% confidence intervals were obtained from the 0.025 and 0.975 percentiles.

### Assessment by whisker spot pattern

Anderson et al. [34] developed a method to identify polar bears based on the pattern of whisker spots on the anterior part of the muzzle. Briefly, processing the images as described by Anderson et al. includes the following steps. (1) Photographs are warped into a standard pixel grid by affine transformation using three spots (corner of the eye, notch of the nose, trailing edge of the mouth) as reference locations. (2) By a series of image adjustments, photographs are enhanced and cropped to arrive at a black-and-white representation of the whisker spot region. (3) The resulting images are compared pairwise on a pixel-by-pixel basis. For all black pixels on photograph 1, the corresponding pixel on photograph 2 is used to calculate the distance to the nearest black pixel on photograph 2. The distances are averaged to arrive at an index of dissimilarity (the Chamfer distance [34]). Similarly, a second index of dissimilarity is calculated from comparing the photographs the other way. Finally, the two estimates are averaged for a measure of dissimilarity between the pair of photographs. We followed the methods described by Anderson et al. [34] with the following modifications. (1) The program was run in a Python environment with ImageMagick (https://www.imagemagick.org) to process the images. (2) After generating a black-and-white representation of the whisker spot area, we filtered unwanted noise from the images by removing isolated black spots that were less than 2 pixels in size. The index of dissimilarity was taken as a starting point for further calculations [34]. A threshold was set such that pairs with dissimilarity below the threshold were rated as being the same, and above the threshold as different. Increasing the threshold caused a drop in the probability that two sets of photographs of the same individual were erroneously rated as different (=FN); however, the probability that sets of different individuals were rated as similar (=FP) increased. The optimal threshold would minimise these two types of errors [43]. For graphic representation, FNR was first plotted against FPR at increasing threshold values in a modified ROC plot [44]. The optimal threshold was found as the minimal distance from any of the points on the curve to the bottom-left corner of the graph (FPR and FNR both zero) using the package pROC in R [44]. Confidence intervals of FNR at any FPR were obtained by bootstrapping with 10,000 replicates [44].

For the automated whisker spot analysis to provide useful results, the photographs should be of sufficient quality [34]. For precise mapping of the spots, the head of the polar bear must be perpendicular to the viewing axis of the camera. Sets in this study in which none of the photographs met these criteria were not used in the analysis. Following Anderson et al. [34] we subjectively qualified photographs as high quality, low quality, or unsuitable (head not in correct position or whisker spots not distinguishable). From each set, two photographs were selected for analysis, one for the left side of the head and one for the right. Subsequently, the pairwise comparisons were separated into a high-quality group (photographs of both sets were of high quality) and a low-quality group (photographs of only one or none of the sets were of high quality). When photographs were available for both sides of the head for both sets, the pair with the lowest dissimilarity index was selected for further analyses.

## Results

The ten observers had equal ratings (i.e. all were “different” or “same”) in 88.6% of the comparisons (n = 525), whereas in the remaining 11.4%, ratings differed to varying degrees. This apparently high degree of consistency in the ratings was confirmed by a repeatability of 0.678 (SE 0.011). Nevertheless, error rates differed widely among observers. A multi-comparison test of the error rates in relation to experience of the test observers revealed a dichotomy, in that observers with experience in photogrammetric techniques performed better than those experienced in observing polar bears or without relevant experience at all (P < 0.001; S1 Table). There was only weak evidence that among the photogrammetry-trained observers, additional experience with polar bears improved the quality of ratings (P = 0.025; S1 Table).

**Fig 3:**
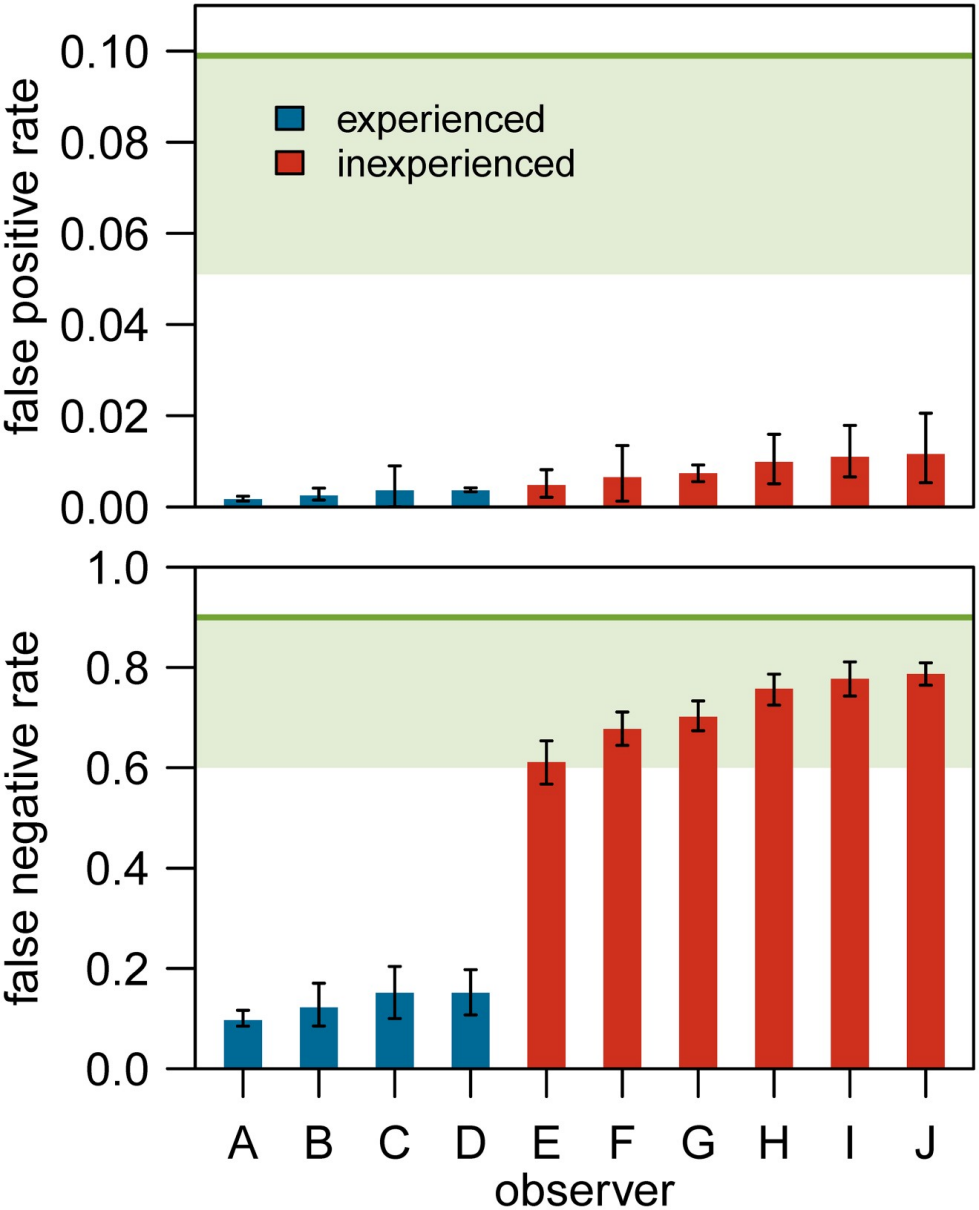
Results of the experiment to identify polar bears from photographs by ten observers (A–J). Shown are the means and 95% confidence intervals for the false positive rates FPR (top panel) and false negative rates FNR (bottom panel), as based on the generalized linear mixed-effects model in Table 3. Observers are ranked by error rates. The horizontal lines represent the mean error rates resulting from simulating a random process. The shaded areas are the lower parts of the 95% confidence intervals. Observers are separated by experience in photogrammetric techniques. Observers A, B, H and I were experienced in observing polar bears.

The final model explored in which way FPR and FNR varied with experience (i.e. experience in photogrammetric techniques) by the inclusion of an interaction between experience and the factor describing whether the same or different bears were compared (similarity class in Table 3). The model results showed that the errors were smaller when different bears were compared than in a comparison between the same individuals (FPR < FNR). Moreover, the significant interaction term indicates that on top of larger errors within the group of inexperienced observers, the errors were particularly large when inexperienced observers compared the same bears (Fig 3, Table 4).

**Table 3:** Modelling the effects of experience and similarity class on error rates in identifying polar bears.

Term	Estimate	SE	z	P
Intercept	-7.938	0.858	-9.244	<0.001
Experience	1.153	0.460	2.50	0.0122
Similarity class	5.486	1.014	5.40	<0.001
Experience × similarity class	2.623	0.608	4.30	<0.001

Data were analysed by a generalized linear mixed-effects model with a binomial distribution and logit link function. Dependent variable is “outcome” indicating whether a rating is correct (0) or not (1). Experience is a factor representing the experience in photogrammetric techniques (0 = experienced, 1 = inexperienced). Similarity class is a factor indicating whether photo sets concerned the same individual (0) or different individuals (1), resulting in estimates of FPR and FNR, respectively. Observer (n = 10) and comparison ID (n = 525) within trial (n = 5) were random factors. Results are on a logit scale.

**Table 4:** Model results comparing the FPR and FNR among experienced (n = 4) and inexperienced (n = 6) observers identifying polar bears.

	Experienced		Inexperienced	
	Mean	SE	Mean	SE
FPR	0.003	0.001	0.009	0.002
FNR	0.130	0.020	0.719	0.018
Error rate	0.130	0.020	0.719	0.018

Estimates are based on the model depicted in Table 3. Experience is by photogrammetric training.

Under a random process, the average expected FNR was 0.901 (95% confidence interval 0.600–1.000), and the average expected FPR was 0.099 (0.051–0.170). All observers had a lower FPR than expected based on a random process, as indicated by the gap between the 95% confidence intervals (Fig 3). Concerning FNR, the four observers experienced in photogrammetric techniques performed better than expected from a random process, whereas the performance of the six inexperienced observers exhibited an overlap with a random process.

**Fig 4:**
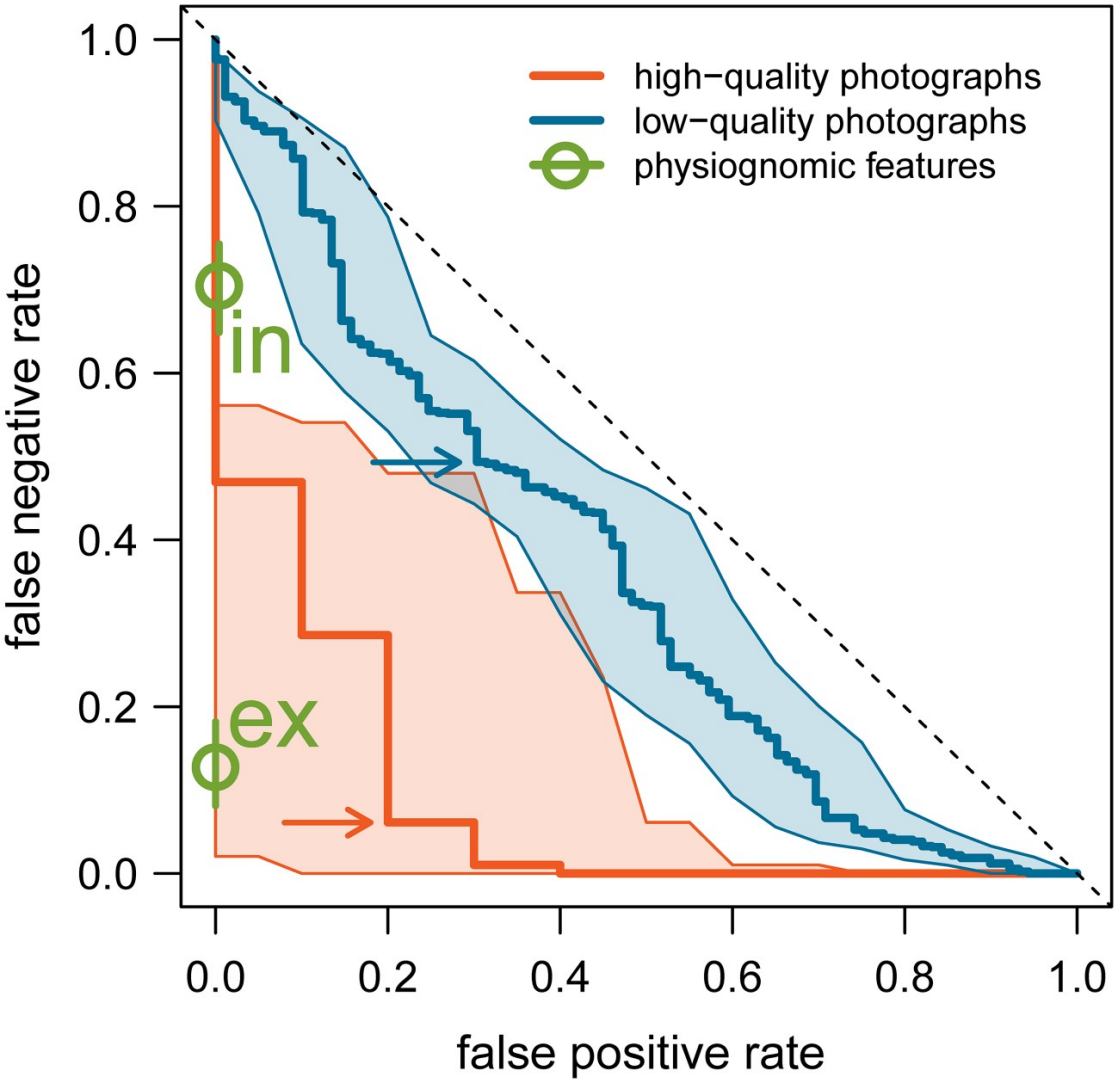
Modified ROC plot of error rates in polar bear identification. Errors comprise false positive rate FPR (different individuals are thought to be the same) and false negative rate FNR (same individuals are not recognised). In the whisker spot pattern analysis, the errors vary as indicated by the solid lines dependent on the chosen threshold of dissimilarity. At a low threshold, FNR is large and FPR is small. Error rates in high-quality photographs are smaller than in low-quality photographs. Shading gives the 95% confidence intervals. Points on the lines resulting in the lowest error rates are indicated by an arrow. The diagonal dashed line represents the relationship when decisions on similarity are at random. For comparison, mean FPR and FNR based on physiognomic features by observers experienced (“ex”) and inexperienced (“in”) in photogrammetric techniques are indicated by the circles (with associated green lines representing the 95% confidence intervals).

In the automated whisker spot analysis, photographs were rated as high-quality in 19 out of 75 sets (a proportion of 0.253) and poor-quality in 31 sets (0.413). In 25 sets (0.333), none of the photographs were adequate to distinguish whisker spots. The number of individual polar bears in the two categories were 9 (high-quality photographs) and 11 (low-quality), respectively. In the comparisons among the high-quality sets, an optimal threshold, which minimises the probability of a mis-classifications, of 2.8 was found for the dissimilarity index. At this optimum, FNR was 0.061 and FPR 0.200, resulting in an error rate of 0.256 (Fig 4). Similarly, when comparing lower-quality photographs, the optimal threshold of the dissimilarity index was 4.0 with an associated FNR of 0.493, FPR of 0.303, and error rate of 0.579 (Fig 4).

For comparison with the automated whisker spot analysis, the FPR and FNR based on physiognomic features are shown in Fig 4. Observers experienced in photogrammetric techniques performed better than the whisker spot analysis of high-quality photographs, though there was an overlap in the 95% confidence intervals. When the whisker spot analysis was based on low-quality photographs, both groups of test observers performed better than the whisker spot analysis.

## Discussion

This study successfully assessed whether polar bears can be individually identified beyond the range of working distances needed when using an established method with whisker spot patterns [24,34]. In contrast to whisker spots (with an ultimate range of 50 m), the physiognomic features used in this study can be distinguished on photographs of wild polar bears at distances of up to 400 m, obtained by standard optical equipment. This means that this technique can be used to study polar bears that are not habituated to the presence of humans and therefore can only be observed from distances that are too far to take adequate photographs for whisker spot analysis.

This study underlines the importance of a training for correct assessments [45]. It was revealing that within our sample of observers, experience with photogrammetric techniques, rather than experience with polar bears by previous intensive observations in the wild, was associated with skills to identify individuals from photographs. Interestingly, observers without photogrammetric experience did almost as well as experienced observers in establishing that two bears were different (the FPRs were only slightly different between the two groups). However, observers without photogrammetric experience fell short in detecting smaller details needed to establish that two bears were the same. Consequently, the FNR of observers without photogrammetric experience exhibited an overlap with results from random simulations.

Any technique to identify individuals in a non-automated way improves when observers acquire the needed skills and get used to individual variation in appearance. Evans [8] describes how observers had to get acquainted to the specific colour pattern on the bill of Bewick’s swans *Cygnus columbianus bewickii* to identify individuals properly. Similarly, processing photographs in an automated way requires trained observers as well, and shifts the labour and required competence to the collecting of high-quality photographs [22,34]. Regardless of what stage of the identification process requires the most effort, the stored photographs give an opportunity to check assessments and, if necessary, to apply corrections with new insights or improved technologies.

With the probability of incorrect assessment being on average 0.13 for observers experienced in photogrammetric techniques, this test supports the supposition that individual polar bears can be recognised reasonably well using physiognomic features. The results indicate that if any error is made, it is most likely that the identity of the same individual is overlooked. Missing a true match is the more common source of errors in other species as well [6,46], which may lead to biased abundance estimates when the observations are used in mark–resight analyses [47]. It is therefore important to reduce identification errors in photographic matching. This reduction can be achieved by first splitting the observations by well-defined grouping variables [48]. A single grouping variable with two levels reduces the number of required comparisons by approximately 50% when groups are similarly sized, and with two grouping variables (yielding 4 combinations) the reduction is 75%. In polar bear field studies, for example, observations can be grouped by gender, age, tagging status (e.g. ear marks or collars) and female breeding status (accompanied by cubs or not), thus potentially reducing the number of pairwise comparisons and identification errors considerably.

Testing for how long distinctive identifying features remain unaltered was beyond the scope of this experimental study. In general, the pattern of natural markers may change over animals’ lifetimes (but see Bauwens et al. [48]), and with longer periods between successive observations, extra care is needed to properly identify an animal to avoid false negatives (i.e. overlooking the return of a familiar animal). This may particularly hold for polar bears, in which structural body growth continues up until the age of four (females) or five (males) years [49].

For several reasons, the method to identify polar bears by whisker spot patterns as developed by Anderson et al. [34] is attractive. First, an objective metric of dissimilarity is obtained. Second, processing photographs in an automated workflow avoids laborious scrutiny of photographs. However, our results indicate that identification by physiognomic features may be more accurate than analysis based on whisker spot patterns. In observers with photogrammetric experience, the probability of an error was on average two times smaller than when analysing by whisker spot patterns, and the difference was even larger when low-quality photographs were used. Despite the considerable difference also when using high-quality photographs, the difference in error rates between the two methods was not statistically significant. We attribute this to a lack of power as the sample size of high-quality photographs was low. In addition, the better performance of the non-automated method may be partly attributed to the fact that our study photographs were taken to distinguish a wide array of physiognomic features and not specifically for whisker spot analysis. Therefore, the quality of the photographs may have fallen short. In their work on whisker spot patterns in polar bears, Anderson et al. [34] achieved more accurate results with their probability of error being tenfold smaller than ours, but this was for excellent photographs, which formed only 10% of the material collected in that study. Error rates associated with the more abundant photographs of sub-excellent quality were in line with our results: 0.16–0.53, as derived from Fig 8 in Anderson et al. [34], versus 0.209–0.579 in this study. Finally, identification by physiognomic characteristics may be more accurate than analysis by patterns of whisker spots because the first uses several keys in the identification process, rather than focusing on a single aspect of an animal’s physiognomy. When photographs are rated as excellent, the method using whisker spot patterns may be the preferred approach. When quality is lower or when bears were photographed at distances larger than 50 m, the method based on a set of physiognomic features gives more accurate results. We suggest, therefore, that wildlife studies may benefit from applying several identification techniques to collect data under different circumstances.

## Supporting information

S1 Fig Example pictures to illustrate which kind of photographs were requested. (PDF)

S1 Dataset Ratings by comparison by observer. (XLSX)

S1 File Script to obtain estimates of FPR and FNR when assuming a random process. (PDF)

S1 Table Model results of exploring effects of experience on error rates. (PDF)

## References

[1] Dell AI, Bender JA, Branson K, Couzin ID, de Polavieja GG, Noldus LPJJ, et al Automated image-based tracking and its application in ecology. Trends Ecol Evol. 2014;29: 417–428. 10.1016/j.tree.2014.05.004 24908439

[2] Möcklinghoff L, Schuchmann K-L, Marques MI. New non-invasive photo-identification technique for free-ranging giant anteaters (*Myrmecophaga tridactyla*) facilitates urgently needed field studies. J Nat Hist. 2018;52: 2397–2411. 10.1080/00222933.2018.1537407

[3] Silvy NJ, Lopez RR, Peterson MJ. Techniques for Marking Wildlife The wildlife techniques manual. Baltimore, Md: Johns Hopkins University Press; 2012.

[4] Pennycuick CJ, Rudnai J. A method of identifying individual lions *Panthera leo* with an analysis of the reliability of identification. J Zool. 1970;160: 497–508. 10.1111/j.1469-7998.1970.tb03093.x

[5] Alonso RS, McClintock BT, Lyren LM, Boydston EE, Crooks KR. Mark-Recapture and Mark-Resight Methods for Estimating Abundance with Remote Cameras: A Carnivore Case Study. PLoS ONE. 2015;10: e0123032 10.1371/journal.pone.0123032 25822245 PMC4378916

[6] Kelly MJ. Computer-aided photograph matching in studies using individual identification: an example from Serengeti cheetahs. J Mammal. 2001;82: 440–449.

[7] Grellier K, Hammond PS, Wilson B, Sanders-Reed CA, Thompson PM. Use of photo-identification data to quantify mother-calf association patterns in bottlenose dolphins. Can J Zool. 2003;81: 1421–1427. 10.1139/z03-132

[8] Evans ME. Recognizing individual Bewick’s Swans by bill pattern. Wildfowl. 1977;28: 153–158.

[9] Sollmann R, Mohamed A, Samejima H, Wilting A. Risky business or simple solution—Relative abundance indices from camera-trapping. Biol Conserv. 2013;159: 405–412. 10.1016/j.biocon.2012.12.025

[10] Pauli JN, Whiteman JP, Riley MD, Middleton AD. Defining Noninvasive Approaches for Sampling of Vertebrates. Conserv Biol. 2010;24: 349–352. 10.1111/j.1523-1739.2009.01298.x 19624526

[11] Barrett MW, Nolan JW, Roy LD. Evaluation of a Hand-Held Net-Gun to Capture Large Mammals. Wildl Soc Bull. 1982;10: 108–114.

[12] Arnemo JM, Ahlqvist P, Andersen R, Berntsen F, Ericsson G, Odden J, et al Risk of capture-related mortality in large free-ranging mammals: experiences from Scandinavia. Wildl Biol. 2006;12: 109–113.

[13] Schemnitz SD, Batcheller GR, Lovallo MJ, White HB, Fall MW. Capturing and Handling Wild Animals The wildlife techniques manual. Baltimore, MD: Johns Hopkins University Press; 2009 pp. 232–269.

[14] Polley L. Navigating parasite webs and parasite flow: Emerging and re-emerging parasitic zoonoses of wildlife origin. Int J Parasitol. 2005;35: 1279–1294. 10.1016/j.ijpara.2005.07.003 16168994

[15] Calvo B, Furness RW. A review of the use and the effects of marks and devices on birds. Ringing Migr. 1992;13: 129–151. 10.1080/03078698.1992.9674036

[16] Regel J, Pütz K. Effect of human disturbance on body temperature and energy expenditure in penguins. Polar Biol. 1997;18: 246–253. 10.1007/s003000050185

[17] Romero LM, Reed JM. Collecting baseline corticosterone samples in the field: is under 3 min good enough? Comp Biochem Physiol A Mol Integr Physiol. 2005;140: 73–79. 10.1016/j.cbpb.2004.11.004 15664315

[18] Walker KA, Trites AW, Haulena M, Weary DM. A review of the effects of different marking and tagging techniques on marine mammals. Wildl Res. 2012;39: 15 10.1071/WR10177

[19] Sandström CAM, Prop J, van der Jeugd H, Loonen MJJE. Baseline Immune Activity Is Associated with Date Rather than with Moult Stage in the Arctic-Breeding Barnacle Goose (*Branta leucopsis*). PLoS ONE. 2014;9: e114812 10.1371/journal.pone.0114812 25517982 PMC4269420

[20] Foster JB. The Giraffe of NairobiI National Park: Home range, sex ratios, the herd, and food. Afr J Ecol. 1966;4: 139–148. 10.1111/j.1365-2028.1966.tb00889.x

[21] Petersen JCB. An identification system for zebra (*Equus burchelli*, Gray). Afr J Ecol. 1972;10: 59–63. 10.1111/j.1365-2028.1972.tb00858.x

[22] Osterrieder SK, Salgado Kent C, Anderson CJR, Parnum IM, Robinson RW. Whisker spot patterns: a noninvasive method of individual identification of Australian sea lions (*Neophoca cinerea*). J Mammal. 2015;96: 988–997. 10.1093/jmammal/gyv102 26937048 PMC4668990

[23] Banerjee K, Jhala YV, Chauhan KS, Dave CV. Living with Lions: The Economics of Coexistence in the Gir Forests, India. PLoS ONE. 2013;8: e49457 10.1371/journal.pone.0049457 23341871 PMC3547023

[24] Anderson CJR, Roth JD, Waterman JM. Can whisker spot patterns be used to identify individual polar bears? J Zool. 2007;273: 333–339. 10.1111/j.1469-7998.2007.00340.x

[25] Timm RM, Baker RO, Bennett JR, Coolahan CC. Coyote attacks: an increasing suburban problem. Proc 21st Vertebr Conf. 2004: 12.

[26] Dubois S, Fraser D. A Framework to Evaluate Wildlife Feeding in Research, Wildlife Management, Tourism and Recreation. Animals. 2013;3: 978–994. 10.3390/ani3040978 26479747 PMC4494361

[27] Stirling I, Derocher AE. Effects of climate warming on polar bears: a review of the evidence. Glob Change Biol. 2012;18: 2694–2706. 10.1111/j.1365-2486.2012.02753.x 24501049

[28] Hamilton SG, Derocher AE. Assessment of global polar bear abundance and vulnerability. Anim Conserv. 2019;22: 83–95. 10.1111/acv.12439

[29] Vongraven D, Aars J, Amstrup S, Atkinson SN, Belikov S, Born EW, et al A circumpolar monitoring framework for polar bears. Ursus. 2012;23: 1–66. 10.2192/URSUS-D-11-00026.1

[30] Rode KD, Pagano AM, Bromaghin JF, Atwood TC, Durner GM, Simac KS, et al Effects of capturing and collaring on polar bears: findings from long-term research on the southern Beaufort Sea population. Wildl Res. 2014;41: 311–322. 10.1071/WR13225

[31] Dyck MG, Baydack RK. Vigilance behaviour of polar bears (*Ursus maritimus*) in the context of wildlife-viewing activities at Churchill, Manitoba, Canada. Biol Conserv. 2004;116: 343–350. 10.1016/S0006-3207(03)00204-0

[32] Prop J, Aars J, Bårdsen B-J, Hanssen SA, Bech C, Bourgeon S, et al Climate change and the increasing impact of polar bears on bird populations. Front Ecol Evol. 2015;3: 33 10.3389/fevo.2015.00033

[33] Pollard KA, Blumstein DT, Griffin SC. Pre-screening acoustic and other natural signatures for use in noninvasive individual identification: Pre-screening natural signatures. J Appl Ecol. 2010;47: 1103–1109.

[34] Anderson CJR, Lobo ND, Roth JD, Waterman JM. Computer-aided photo-identification system with an application to polar bears based on whisker spot patterns. J Mammal. 2010;91: 1350–1359. 10.1644/09-MAMM-A-425.1

[35] Stirling I, Thiemann GW, Richardson E. Quantitative support for a subjective fatness index for immobilized polar bears. J Wildl Manag. 2008;72: 568–574. 10.2193/2007-123

[36] Fawcett T. An introduction to ROC analysis. Pattern Recognit Lett. 2006;27: 861–874. 10.1016/j.patrec.2005.10.010

[37] R Core Team. R: A language and environment for statistical computing. R Foundation for Statistical Computing Vienna, Austria: R Foundation for Statistical Computing; 2019 http://www.R-project.org/

[38] Bates D, Mächler M, Bolker B, Walker S. Fitting linear mixed-effects models using lme4. J Stat Softw. 2015;67: 1–48. 10.18637/jss.v067.i01

[39] Koo TK, Li MY. A Guideline of Selecting and Reporting Intraclass Correlation Coefficients for Reliability Research. J Chiropr Med. 2016;15: 155–163. 10.1016/j.jcm.2016.02.012 27330520 PMC4913118

[40] Stoffel MA, Nakagawa S, Schielzeth H. rptR: repeatability estimation and variance decomposition by generalized linear mixed-effects models. Methods Ecol Evol. 2017;8: 1639–1644. 10.1111/2041-210X.12797

[41] Lenth R. emmeans: Estimated marginal means, aka Least-squares Means. R-package version 1.4.1. Aka Least-squares Means, R. 2019. https://github.com/rvlenth/emmeans

[42] Canty A, Ripley B. boot: Bootstrap R (S-Plus) functions. R package version 1.3–23. 2019. https://cran.r-project.org/web/packages/boot/index.html

[43] Pollack I. A nonparametric procedure for evaluation of true and false positives. Behav Res Methods Instrum. 1970;2: 155–156. 10.3758/BF03209289

[44] Robin X, Turck N, Hainard A, Tiberti N, Lisacek F, Sanchez J-C, et al pROC: an open-source package for R and S+ to analyze and compare ROC curves. BMC Bioinformatics. 2011;12: 77 10.1186/1471-2105-12-77 21414208 PMC3068975

[45] Lahiri M, Tantipathananandh C, Warungu R, Rubenstein DI, Berger-Wolf TY. Biometric animal databases from field photographs. ACM Press; 2011 10.1145/1991996.1992002

[46] Stevick PT, Palsbøll PJ, Smith TD, Bravington MV, Hammond PS. Errors in identification using natural markings: rates, sources, and effects on capture-recapture estimates of abundance. Can J Fish Aquat Sci. 2001;58: 1861–1870.

[47] Urian K, Gorgone A, Read A, Balmer B, Wells RS, Berggren P, et al Recommendations for photo-identification methods used in capture-recapture models with cetaceans. Mar Mammal Sci. 2015;31: 298–321. 10.1111/mms.12141

[48] Bauwens D, Claus K, Mergeay J. Genotyping validates photo-identification by the head scale pattern in a large population of the European adder (*Vipera berus*). Ecol Evol. 2018;8: 2985–2992. 10.1002/ece3.3917 29531711 PMC5838086

[49] Lønø O. The polar bear (*Ursus maritimus* Phipps) in the Svalbard area. Nor Polarinst Skr. 1970;149: 1–103.

