# Supplementary figures and images for: Identifying individual polar bears at safe distances: A test with captive animals
Source: PLoS One. 2020 Feb 13;15(2):e0228991. doi: 10.1371/journal.pone.0228991 (PMC7018014; doi:10.1371/journal.pone.0228991)

A

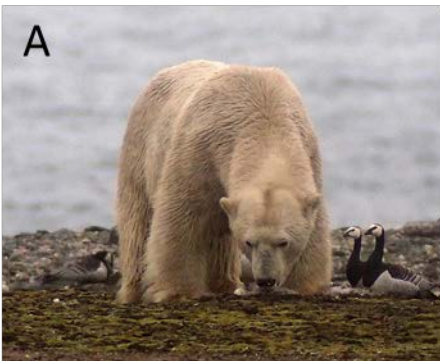

A) Front view top head

B) Front view right

C) Front view left

D) Left view

E) Right view

B

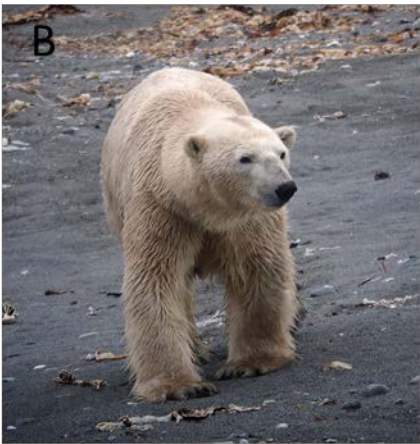

C

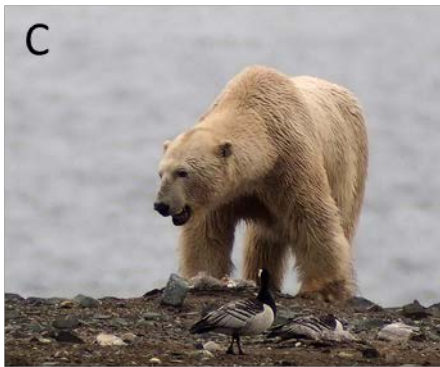

D

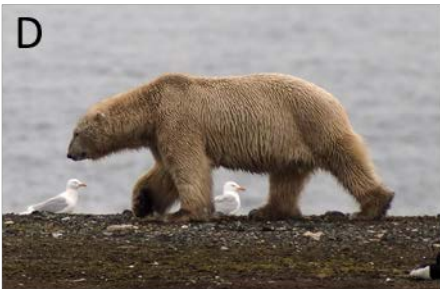

E

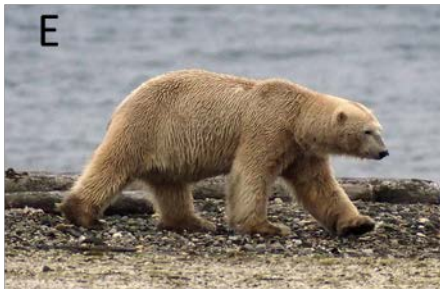

Supplement: S1 Fig — (PDF) [file pone.0228991.s001.pdf]
