# Supplementary material for: Identifying individual polar bears at safe distances: A test with captive animals
Source: PLoS One. 2020 Feb 13;15(2):e0228991. doi: 10.1371/journal.pone.0228991 (PMC7018014; doi:10.1371/journal.pone.0228991)
Supplement: S1 File — (PDF) [file pone.0228991.s003.pdf]

```
# Python (3.6.4) script to calculate FPR and FNR under a random process.
# Output need to be analysed for means and confidence intervals.
import random
import numpy
k=0
l=0
j=0
setbear2=[]
fileOutput='d:/fileOut.txt'
# initialise
oorspr = numpy.zeros((15, 15))
testset = numpy.zeros((15, 15))
# These are the individual IDs for one of the trials
setbear=[13,3,13,11,6,12,9,2,1,5,12,7,3,5,6]

# 1000 simulations and write results to output file
for Ronde in range (0,1000):
    # compare base-record with random order
    # 1= create a random series
    setbear2=[random.randrange(1, 11) for _ in range(0, 15)]
    truepositive=0
    truenegative=0
    falsepositive=0
    falsenegative=0
    cond1=0
    cond2=0
    cond3=0
    cond4=0
    # read the base series
    for i in range (0,15):
        for j in range (i+1,15):
            if setbear[i]==setbear[j]:
                oorspr[i,j]=1
            else:
                oorspr[i,j]=0
    # read the testset
    for i in range (0,15):
        for j in range (i+1,15):
            if setbear2[i]==setbear2[j]:
                testset[i,j]=1
            else:
                testset[i,j]=0
    # compare the base series with the testset
    for i in range (0,15):
        for j in range (i+1,15):
            cond1 = oorspr[i,j] == 1 and testset[i,j] == 1
            cond2 = oorspr[i,j] == 1 and testset[i,j] == 0
            cond3 = oorspr[i,j] == 0 and testset[i,j] == 1
            cond4 = oorspr[i,j] == 0 and testset[i,j] == 0
            if cond1 == 1:
                truepositive=truepositive+1
            elif cond2==1:
                falsenegative=falsenegative+1
            elif cond3==1:
                falsepositive=falsepositive+1
            elif cond4==1:
                truenegative=truenegative+1
    with open(fileOutput,'a') as f:
                f.write(str(truepositive)+", ")
                f.write(str(truenegative)+", ")
                f.write(str(falsepositive)+", ")
                f.write(str(falsenegative))
                f.write("\n")
```
