# Supplementary material for: Identifying individual polar bears at safe distances: A test with captive animals
Source: PLoS One. 2020 Feb 13;15(2):e0228991. doi: 10.1371/journal.pone.0228991 (PMC7018014; doi:10.1371/journal.pone.0228991)
Supplement: S1 Table — (PDF) [file pone.0228991.s004.pdf]

S1 Table. Comparison of ratings by observers with experience varying by studying polar bears (pb) and photogrammetric techniques (pht). Results of a generalized linear mixed-effects model with a binomial distribution and logit link function. Fixed factors were experience type and similarity class (whether bears were the same or not). Observer (n = 10) and comparison ID (n = 525) within trial (n = 5) were random factors. Experience types are coded as: 1 = experience by pb and pht, 2 = experience by pb, 3 = experience by pht, 4 = no experience. Given are (A) the estimated marginal means EMM and SE on the logit scale with results averaged over similarity class, and (B) the comparisons between the types, adjusted by the Tukey method for multiple comparisons (on the log odds ratio scale).

(A)

Types of experience	EMM	SE
1	–6.41	0.732
2	–2.27	0.434
3	–4.6	0.54
4	–2.84	0.425

(B)

Types compared	Estimate	SE	z	P
1–2	–4.148	0.636	–6.522	<0.001
1–3	–1.812	0.643	–2.82	0.025
1–4	–3.575	0.606	–5.902	<0.001
2–3	2.336	0.43	5.426	<0.001
2–4	0.573	0.294	1.953	0.206
3–4	–1.763	0.392	–4.492	<0.001
